# Pooling the evidence: A review of swimming and atopic dermatitis

**DOI:** 10.1111/pde.15325

**Published:** 2023-04-07

**Authors:** Cathal O'Connor, Siobhan McCarthy, Michelle Murphy

**Affiliations:** ^1^ Dermatology South Infirmary Victoria University Hospital Cork Ireland; ^2^ Paediatrics and Child Health Cork University Hospital Cork Ireland; ^3^ Medicine University College Cork Cork Ireland; ^4^ INFANT Research Centre University College Cork Cork Ireland

**Keywords:** atopic dermatitis, atopy, exercise, quality of life, swimming

## Abstract

Swimming is an excellent form of aerobic exercise and is an essential life skill. Many children with atopic dermatitis (AD) are advised not to swim because of concerns about negative impacts on their skin disease, and some children with AD do not swim because they are self‐conscious about the appearance of their skin. We aimed to perform a narrative review of the available literature on swimming and AD and scientifically analyze the potential impact of all components of swimming in AD—water, skin barrier, swimming gear, and exercise. Studies examined the impact of swimming on the skin barrier and the relative contraindications to swimming. Constituents of water which may affect AD include hardness, pH, temperature, antiseptics, and other chemicals. Potential interventions to reduce damage included emollient application, special swim gear, and showering post‐submersion. The benefits of swimming as a form of exercise in AD included reduced sweating, cardiorespiratory fitness, and maintenance of healthy weight. Drawbacks of swimming as a form of exercise in AD included the limited benefit on bone mineral density. Future research should examine the impact of swimming on flares of AD using noninvasive biomarkers as well as clinical severity assessment and assess the role for different types of emollient as an intervention for optimal eczema control. This review highlights gaps in the scientific literature on swimming and AD and provides evidence‐based guidance on interventions to minimize deleterious effects on skincare and maximize opportunities for children with AD to swim.

## 1 INTRODUCTION

**FIGURE 1:**
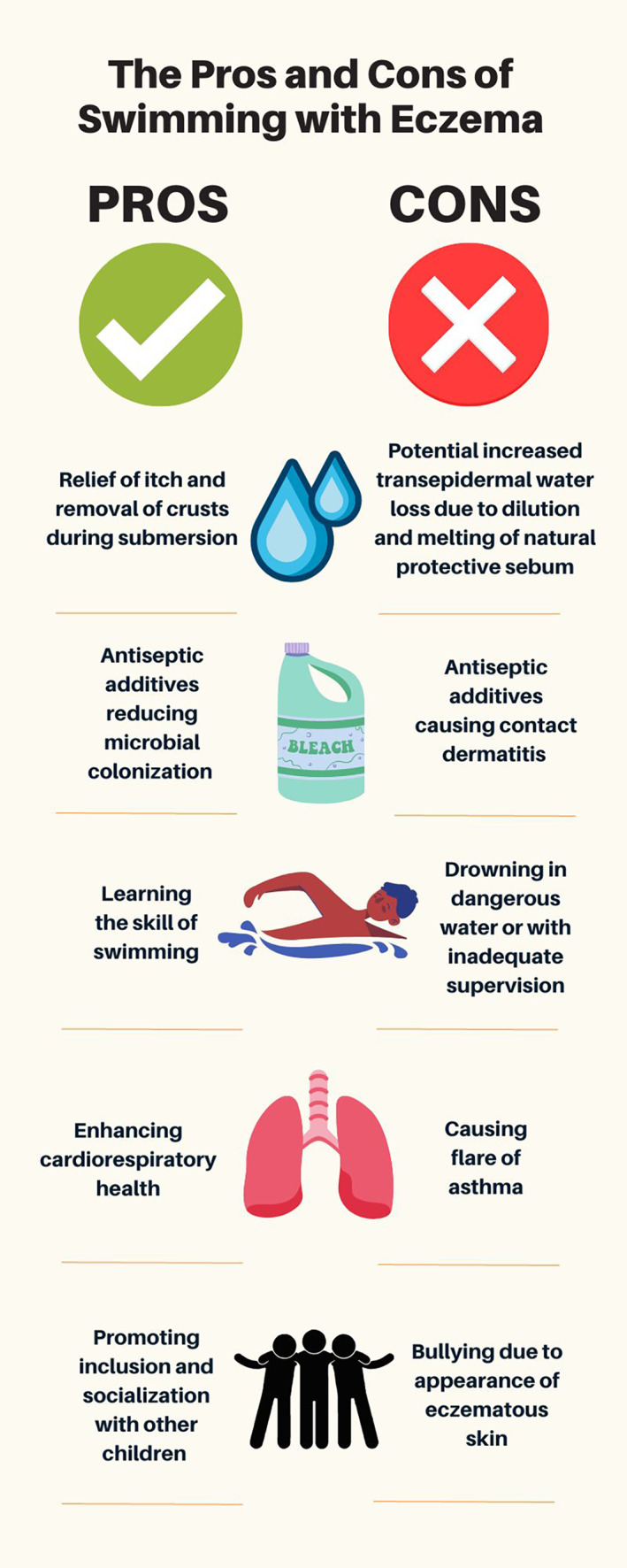
Potential benefits and risk of swimming for children with atopic dermatitis.

Swimming in childhood is known to be associated with enhanced physical and mental health,^1^ as well as being an essential life skill to reduce the risk of drowning.^2^ In particular, outdoor swimming has dramatically increased in popularity during the COVID‐19 pandemic, with myriad reported health benefits.^3^ Swimming is a suitable choice of exercise for children with atopic dermatitis (AD), as the water contact can be cooling for exposed eczematous skin, and excessive sweating is avoided. However, up to a third of children with AD have disrupted access to swimming due to the severity of their disease.^4^ Some children with AD are advised not to swim due to concerns about potential negative effects on their skin, and some children may be reluctant to swim due to self‐consciousness about the visual impact of their eczematous skin in front of others. Potential benefits and risks of swimming for children with AD are outlined in Figure 1.

AD may have a profoundly negative effect on health‐related quality of life (QOL).^5^ Key factors in reduced QOL are lifestyle restrictions or limitations imposed because of AD. Prohibiting children with AD from swimming can lead to deterioration in both physical and mental health. Exclusion from peer activities such as swimming can lead to “othering” of children with AD, and put them at risk of teasing, bullying, and further emotional upset.^6^ Subsequent inability to swim can lead to future embarrassment, and increase the risk of drowning, which is the third leading cause of premature death globally, with children accounting for the highest rate (https://www.who.int/news-room/fact-sheets/detail/drowning).

This narrative review aims to synthesize the currently available evidence on swimming and AD, and analyze the components of water, skin barrier protection, swimming gear, and exercise that may be relevant to children with AD. The content of the review is used to formulate guidance for dermatologists and pediatricians on what advice to provide to children with AD regarding swimming.

## 2 METHODS

We performed a narrative review by searching PubMed for English language articles published up until March 10, 2023, including the terms “atopic dermatitis” OR “atopic eczema” OR “eczema” AND “swimming.” This resulted in 46 unique articles which were then screened by title and abstract for pertinence, resulting in 20 articles that were read in full by all authors. Further targeted reviews were performed to identify research on basic science related to the skin barrier and swimming.

## 3 RESULTS

### 3.1 Current evidence on swimming and AD

One of the key concerns relating to swimming exposure and impact on AD development and severity is the effect of swimming on the skin barrier, which is known to be a primary pathogenic factor in the development and persistence of AD.^7^ Transepidermal water loss (TEWL) is a surrogate marker for skin barrier function, and aberrations in TEWL are known to predate the clinical development of AD.^8^ One study in elite teenage swimmers without AD has shown TEWL increases immediately following 2 h of intensive swimming training, but returns to normal after 30 min.^9^ However, most children are unlikely to spend 2 h in the pool, and TEWL rapidly returns to normal levels after submersion, which is reassuring.

Recently, there has been keen interest in skin barrier‐focused interventions for the prevention of AD in early life, with mixed results.^10^ The effect of infant swimming and emollient application versus swimming alone was examined in a German study in 2009.^11^ Children with a personal or parental history of AD were excluded. Both groups went swimming for 25–40 min weekly for 4 weeks (*n* = 44), and the intervention limb (*n* = 20) applied an emollient after swimming. The intervention group had stable sebum and pH 1 week following the final swim, while a significant decrease in sebum and pH was noted in the group that was not moisturized after swimming.

A Spanish birth cohort study examined the impact of indoor and outdoor swimming pool attendance and eczema development during the first year of life.^12^ When adjusted for parental atopy, type of swimming pool, cumulative duration of pool exposure, and socioeconomic group, there was no difference in AD prevalence in those who were exposed to swimming in the first year of life and those who were not.

Longitudinal studies such as the Avon Longitudinal Study of Parents and Children cohort study^13^ have failed to show an association between early life exposure to swimming and subsequent development of AD. A birth cohort study from Germany showed no increased rates of AD by 6 years in children with early swimming pool attendance.^14^ Another German study that asked adults to retrospectively report childhood exposure to swimming pools showed no correlation between early swimming and lifetime development of AD,^15^ with the significant limitations of recall bias and lack of objectively diagnosed AD.

One Spanish study showed a 40% increased risk of AD development in children who swam before the age of 3 years and a higher risk of AD was observed within the highest tercile of years in swimming practice.^16^ However, AD was self‐reported, and adjustment for parental socioeconomic group and educational status, factors known to be associated with an increased risk of AD in children,^17^ was not performed. Another Spanish study reported increased risk of AD in children who swam in pools before 2 years of age,^18^ but AD was parent‐reported and the possibility of reverse causation cannot be ruled out. Another study looked at current exposure to swimming and AD outcomes,^19^ with no association between current swimming and AD prevalence. However, the authors noted their results may be skewed because children with AD may avoid indoor swimming pools because of potential negative effects on their eczema.

Given parental concerns about potential topical corticosteroid side effects and misinformation related to the treatment of eczema,^20^ parents may seek complementary and alternative medicine (CAM) therapies for AD. One systematic review and meta‐analysis of CAM for treatment of AD showed that children randomized to swimming as therapy improved compared to standard clinical care.^21^ However, the methodological quality of these studies was of unclear or high risk of bias in general, and other CAM therapies in the trials included unproven interventions.

One exploratory study, which closely followed up 60 children with AD for environmental exposures, did not show a link between swimming and daily “bother” score, but did show an association with higher “scratch” scores and “step up treatment” scores.^22^ However, there was no objective assessment of AD severity, and increased “step up treatment” may relate to increased prophylactic emollient usage around the time of swimming. There is no high‐quality evidence available related to swimming exposure and AD outcomes during flares of AD, and this should be explored as a future interventional study. Given the potential for aggravation of skin barrier dysfunction during a flare of AD, it is reasonable to recommend delaying swimming during severe flares. Proactive, rather than reactive, treatment of AD should be optimized to treat disease flares and reduce the frequency of exacerbations.^23^

As there is a risk of transmission of bacteria, particularly of contagious staphylococcal species, during periods of impetiginization of AD, it may also be reasonable to delay swimming during infective flares until weeping or crusting has resolved. One Italian guideline recommends avoiding swimming until 24 h after antimicrobial therapy has been initiated and crust has lifted.^24^ However, chlorinated water has antiseptic properties (as discussed below), so swimming during infective flares may have beneficial effects, which must be weighed against the risk of spreading contagion, for example, via shared towels. In addition, studies in remote Australian indigenous communities have shown that the installation of community swimming pools in fact reduces the prevalence of impetigo and other skin infections.^25^ Molluscum contagiosum (MC), a common pox virus that affects the skin, occurs more frequently both in children with AD and in children without AD who swim regularly.^26^ The potential risk of developing MC, a benign and self‐limiting infection, should not exclude children with AD from swimming. If children with AD develop MC, swimming is still permissible, but it is advisable to cover lesions with a waterproof plaster and avoid sharing towels.

### 3.2 Water constituents

#### 3.2.1 Hardness

Water hardness is defined as the concentration of divalent metal cations, such as dissolved calcium (Ca^++^) and magnesium (Mg^++^) in a water sample. Calcium chloride is added to pools to keep the water hard. This protects the surfaces of the pool from corrosion. However, calcium in hard water may damage the skin barrier, increasing skin dryness and irritation, and both predispose children to AD and provoke flares of established AD.^27^ Skin contact with hard water has been associated with increased TEWL, particularly in patients with AD and filaggrin (*FLG*) mutations.^28^ The interaction between hard water, infant swimming, and AD outcomes has also been examined,^29^ with a linear relationship between water hardness and AD prevalence, and no independent relationship between infant swimming and AD, although the combination of hard water and infant swimming was synergistic in increasing AD prevalence. However, randomized controlled trials comparing water softeners with standard care have not shown a significant difference in objective AD severity with softened water.^27^

#### 3.2.2 pH

pH is the most important factor in swimming pool water chemistry since it affects chlorine efficacy as well as overall balance in the water. Pool water must meet stringent quality standards to provide a healthy experience for swimmers, including protection against the chlorine‐resistant pathogen *Cryptosporidium* (https://www.pwtag.org/). The ideal pH value to be comfortable for eyes and to prevent corrosive or scale‐forming conditions is at a slightly alkaline value in the range of pH 7.2–7.4, while skin on most parts of the body has a pH level that normally ranges from 4.1 to 5.8.^30^ The acid mantle of the skin is responsible for retaining moisture and essential lipids, and providing a barrier against pathogens, irritants, and allergens. Excessive alkalinization of the skin may cause the skin to become dry and irritated, potentially leading to AD.

#### 3.2.3 Temperature

Children with eczema may prefer lower pool temperatures (https://nationaleczema.org/blog/swimming-eczema/), and warmer temperatures may increase TEWL and skin pH.^31^

#### 3.2.4 Antiseptics and other chemicals

Pool water contains multiple chemicals (https://www.pwtag.org/) that may dry out or irritate eczematous skin. Chlorine is added to pools for antiseptic activity, but chlorine exposure can cause irritant contact dermatitis (“pool dermatitis”) or allergic contact dermatitis (“pool water dermatitis”).^32^ However, the antiseptic activity of diluted chlorine may reduce microbial colonization and may reduce AD severity, as described with bleach baths.^33^ Alternative or additional options for disinfecting swimming pools include salt‐water chlorination, bromine, ozone, *polyhexamethylene biguanide, algaecides, and filter aids, flocculants, and clarifiers*.

#### 3.2.5 Seawater/saltwater

Anecdotally, many patients with AD report benefit from swimming in seawater or saltwater pools, while some report irritation. Despite salt bathing being a common practice in AD, good‐quality evidence is lacking on the effect of salt water on AD outcomes. One small Japanese study showed some benefit from balneotherapy with natural mineral dissolved water.^34^ Some studies have reported strong associations between marine water contact and staphylococcal skin infections.^35^

### 3.3 Skin barrier protection while swimming

#### 3.3.1 Emollient barrier

Few studies have examined the benefit of emollient application prior to or after swimming. Garcia Bartels et al. showed persistent enhancement of the cutaneous barrier in infants with emollient application immediately following swimming.^11^ Future research should examine the impact of application of different emollients before and after swimming on transcutaneous biomarkers in children with AD, as well as clinical severity scoring. Children may tolerate emollients with much higher lipid content during submersion than they would otherwise.

### 3.4 Swimming gear and AD

#### 3.4.1 Residual chlorine/irritants

It has been shown that free residual chlorine in bathing water reduces the water‐retaining properties of the stratum corneum in AD.^36^ If swim wear is not removed quickly following submersion, occlusion, and direct irritation can occur quickly, also predisposing children to folliculitis and bacterial infections.^32^ It is important to remove all swim wear quickly following exit from the pool, followed by rinsing in fresh unchlorinated water (if available). If swimming at the beach parents could consider bringing a container of fresh water to rinse off after exiting the seawater.

#### 3.4.2 Contact dermatitis to swim gear

Contact with certain swim gear like goggles, scuba masks, and bathing caps can cause irritation or can sensitize children with AD to various rubber constituents, which may require patch testing for confirmation of allergic contact dermatitis.^32^

### 3.5 Exercise in AD

There is growing awareness of the associations between AD and obesity and cardiovascular disease.^37^ It is important for dermatologists to encourage children with AD to foster healthy lifestyles which will enhance their lifelong well‐being. Sleep disturbance is a cardinal feature of AD, and exercise such as swimming is a valid nonpharmacologic treatment for sleep disruption.^38^ Moreover, swimming is a form of exercise that is associated with less perceptible sweating, which is advantageous for children with AD who have significant heat intolerance. Importantly, given the strong link between AD and asthma, there is no strong evidence showing that swimming exposure causes subsequent asthma development or exacerbation. As swimming is a low‐contact and nonweight bearing form exercise with minimal benefit on bone mineral density,^39^ it is important to consider other weight‐bearing forms of exercise in conjunction with swimming, as patients with AD are known to have an increased risk of osteoporosis.^40^

## 4 ADVICE FOR SWIMMING FOR CHILDREN WITH AD

**FIGURE 2:**
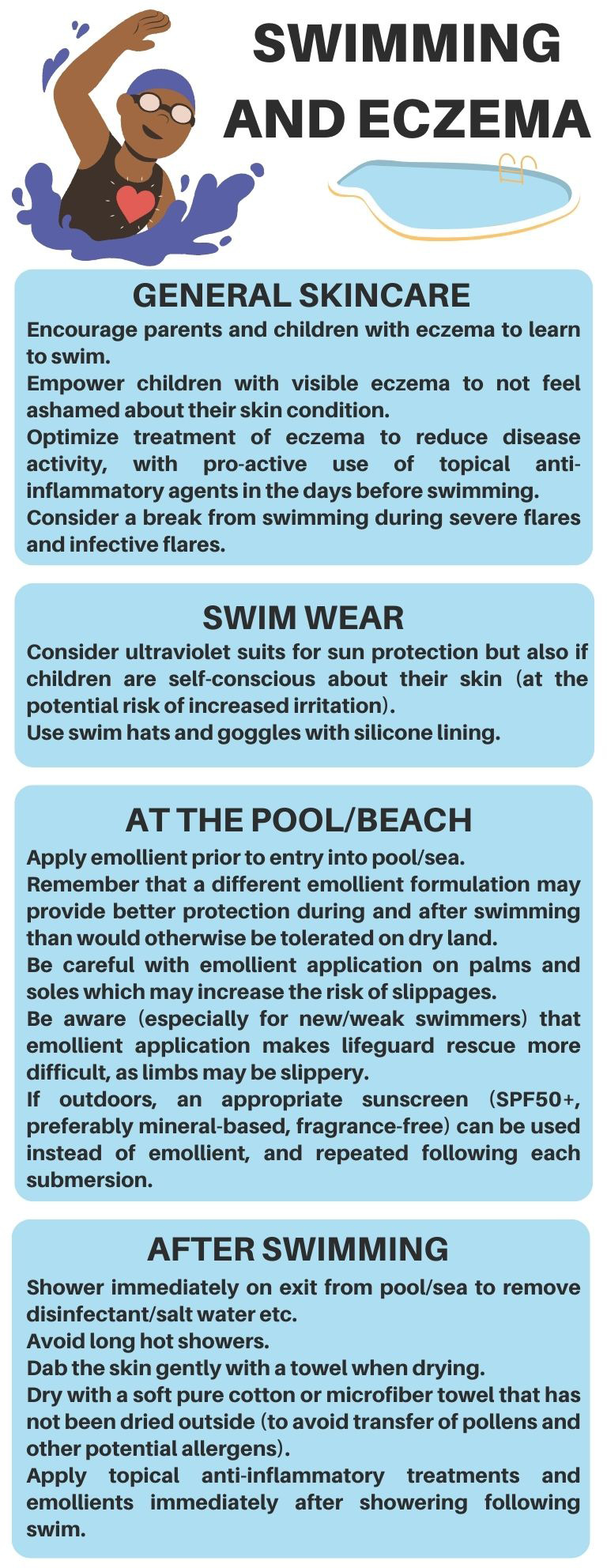
Recommendations from the authors for swimming for children with atopic dermatitis.

Current guidance from eczema support organizations for swimming for children with AD is highlighted in Table 1. Following the extensive literature review on the relationship between swimming and AD, we have formulated the package of advice seen in Figure 2. The following advice is based on a combination of the currently available scientific evidence, and the opinion of the authors.

**TABLE 1:** Advice from patient information leaflets and websites from major patient organizations and dermatology associations (https://nationaleczema.org, https://eczema.org, https://www.aad.org, https://www.bad.org.uk).

Organization	Advice
Eczema Foundation	Apply emollient before swimming^a^ Rinse skin post swimming and pat dry^a^ Wash at home with shower gel containing copper and zin Apply emollient ± topical corticosteroid^a^
National Eczema Society	Avoid swimming if eczema is flaring badly or infected If swimming indoors, apply emollient (preferably ointment) liberally before entering pool^a^ If swimming outdoors, apply sunscreen (preferably containing titanium and/or zinc oxide) Shower immediately after swimming using an emollient and re‐apply emollient^a^ Do not hang around the pool after as skin is still exposed to chlorinated fumes Consider wearing UV‐protection swim suits/clothing for self‐conscious children If pool water at one pool is irritating, avoid swimming immediately after chlorine is added to that pool, try other pools or a salt‐water pool, or try swimming in fresh water or sea water At a new pool, try a short test period to assess your skin's response to the water
American Association of Dermatology	Apply moisturizer before swimming^a^ Rinse the skin with warm water after swimming^a^ Immediately pat the skin dry after rinsing and apply moisturizer^a^
British Association of Dermatology	Rinse well after swimming and apply plenty of moisturizer after drying^a^ Make sure the shower at the swimming pool contains fresh water and not chlorinated water from the swimming pool

^a^ Advice based on clinical or scientific studies.

## 5 CONCLUSION

Swimming is an essential life skill, and children with AD should not miss out on this enjoyable hobby and exercise because of unfounded concerns. Our work identifies significant gaps in the literature on the optimal advice on swimming for children living with AD but provides suggestions for providers based on current evidence and our opinions when evidence was lacking.

Future research should examine the impact of swimming on flares of AD using clinical severity assessment and noninvasive biomarkers and assess the role of different interventions to optimize eczema control when swimming.

## AUTHOR CONTRIBUTIONS

Cathal O'Connor identified the literature gap on the topic, reviewed the literature, wrote the manuscript, and reviewed the manuscript. Siobhan McCarthy and Michelle Murphy reviewed the manuscript and provided feedback. Siobhan McCarthy produced a patient information leaflet based on the literature review.

## FUNDING INFORMATION

Dr Cathal O'Connor is funded by the Irish Clinical Academic Training (ICAT) program, supported by the Wellcome Trust and the Health Research Board (grant number 223047/Z/21/Z); the Health Service Executive National Doctors Training and Planning; and the Health and Social Care, Research and Development Division, Northern Ireland.

## CONFLICT OF INTEREST STATEMENT

The authors declare no conflicts of interest.

## Data Availability

Data derived from public domain resources.
